# Optimisation and Calibration of Bayesian Neural Network for Probabilistic Prediction of Biogas Performance in an Anaerobic Lagoon

**DOI:** 10.3390/s24082537

**Published:** 2024-04-15

**Authors:** Benjamin Steven Vien, Thomas Kuen, Louis Raymond Francis Rose, Wing Kong Chiu

**Affiliations:** 1 Department of Mechanical and Aerospace Engineering, Monash University, Wellington Rd, Clayton, VIC 3800, Australia; wing.kong.chiu@monash.edu; 2 Melbourne Water Corporation, 990 La Trobe Street, Docklands, VIC 3008, Australia; thomas.kuen@melbournewater.com.au; 3 Defence Science & Technology Group, Fishermans Bend, VIC 3207, Australia; francis.rose@defence.gov.au

**Keywords:** deep learning, machine learning, Bayesian neural network, anaerobic lagoon, long short-term memory, mixture density, Monte Carlo dropout, model calibration, hyperparameter optimisation

## Abstract

This study aims to enhance diagnostic capabilities for optimising the performance of the anaerobic sewage treatment lagoon at Melbourne Water’s Western Treatment Plant (WTP) through a novel machine learning (ML)-based monitoring strategy. This strategy employs ML to make accurate probabilistic predictions of biogas performance by leveraging diverse real-life operational and inspection sensor and other measurement data for asset management, decision making, and structural health monitoring (SHM). The paper commences with data analysis and preprocessing of complex irregular datasets to facilitate efficient learning in an artificial neural network. Subsequently, a Bayesian mixture density neural network model incorporating an attention-based mechanism in bidirectional long short-term memory (BiLSTM) was developed. This probabilistic approach uses a distribution output layer based on the Gaussian mixture model and Monte Carlo (MC) dropout technique in estimating data and model uncertainties, respectively. Furthermore, systematic hyperparameter optimisation revealed that the optimised model achieved a negative log-likelihood (NLL) of 0.074, significantly outperforming other configurations. It achieved an accuracy approximately 9 times greater than the average model performance (NLL = 0.753) and 22 times greater than the worst performing model (NLL = 1.677). Key factors influencing the model’s accuracy, such as the input window size and the number of hidden units in the BiLSTM layer, were identified, while the number of neurons in the fully connected layer was found to have no significant impact on accuracy. Moreover, model calibration using the expected calibration error was performed to correct the model’s predictive uncertainty. The findings suggest that the inherent data significantly contribute to the overall uncertainty of the model, highlighting the need for more high-quality data to enhance learning. This study lays the groundwork for applying ML in transforming high-value assets into intelligent structures and has broader implications for ML in asset management, SHM applications, and renewable energy sectors.

## 1. Introduction

The use of artificial intelligence is expected to become more prevalent in the future, including in engineering fields where it has been utilised for predictive maintenance and has enabled scheduling of maintenance [1,2,3,4,5,6]. The core concept of deep learning involves learning hierarchical representations of data through the use of multiple hidden layers. One of the advantages of deep learning is its ability to automatically extract relevant features within these hidden layers of the model, thereby eliminating the need for manual feature engineering. Classical models, such as autoregressive integrated moving averages, have traditionally been used for time series forecasting and are still widely used. However, these models often tend to oversimplify problems, fall short in capturing nonlinearity, and are heavily dependent on predefined assumptions, which may not accurately capture the intricacies and account for external and new factors present in the real world. By contrast, data-driven algorithms have emerged as more prevalent and adaptable approaches, particularly for highly complex systems, where the limitations of such classical models are more apparent.

Recently, there has been considerable interest in employing machine learning (ML) methods in the field of engineering [2,3,4,5,6,7,8], including the effectiveness of probabilistic artificial neural networks (ANNs) in structural health monitoring (SHM) applications. The ability to quantify uncertainty is crucial for engineering prediction systems, as it provides insight into the reliability of their forecasts and, in turn, the level of confidence required for quantitative risk assessment. The adoption of a probabilistic approach to ML enables the measurement of various sources of uncertainties, including those arising from data and the lack of data. Bayesian neural networks (BNNs) are a type of ANN architecture that incorporate Bayesian statistical principles to estimate uncertainty [9,10]. Recently, BNNs have gained significant attention due to their ability to provide uncertainty estimates in a wide range of engineering applications, including SHM [6,7,8]. For instance, Arangio and Bontempi [8] demonstrated BNNs to train on acceleration responses for SHM of a large bridge, achieving an average of 4.0% relative error in modal frequencies compared to finite element (FE) modelling. Additionally, Vega and Todd [7] demonstrated the use of variational inference for BNNs as a surrogate degradation model for miter gates, which reduced maintenance costs by up to 35.1% and resulted in more cost-effective decision making. Nevertheless, it is evident that research on the probabilistic approach for ANN continues to be actively pursued, particularly because of its ability to quantify confidence. This approach is highly recognised for its relevance in numerous applications, as it enhances risk management by accounting for uncertainties in both data and models.

The efficient learning and performance of ML models are greatly influenced by their architectural design and the careful selection of hyperparameters. In ML, hyperparameters are external configurations for a model, such as the number of hidden layers and learning rate, that are not learned from the data but are set prior to model training. Techniques for hyperparameter optimisation, such as grid search and random search [11,12], are employed to optimise computational cost and the learning rate during training as well as enhance the model’s ability to accurately generalise to unseen data. Additionally, probabilistic ANNs often produce overly confident predictions, characterised by narrow confidence intervals that fail to accurately encompass the true data distribution [13,14,15]. This issue often arises from the fact that the optimisation of loss functions primarily measures the goodness of fit rather than uncertainty estimations [15,16,17,18,19]. An ideally well-calibrated model should generate prediction intervals that accurately encompass the correct proportions of observations. For instance, actual values should fall within a 95% prediction interval 95% of the time. Various methods have been developed for calibrating probabilistic ML models quantitatively, including techniques that average and maximise calibration errors, yielding a single scalar metric for both classifications [15], and, more recently, regression models [17,19]. Recent studies [20,21] have incorporated model calibration into their BNNs to ensure accurate uncertainty quantification. Okte and Al-Qadi [21] developed a 3D FE surrogate model using a dropout-based BNN and demonstrated how varying dropout rates influence model uncertainties through calibration curve plots. Nevertheless, it is imperative to emphasise the importance of both hyperparameter optimisation and model calibration in ensuring the accuracy and reliability of probabilistic ANNs for practical applications.

**Figure 1:**
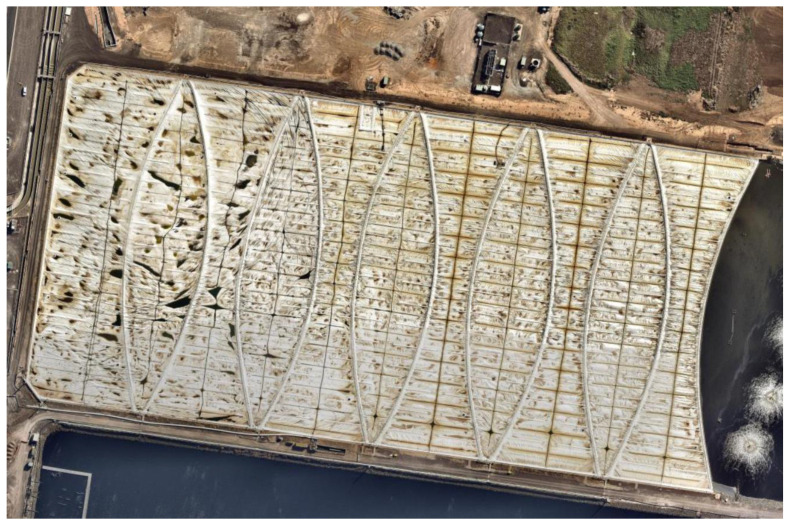
Aerial view of 55E anerobic lagoon at Melbourne Water’s WTP.

Melbourne Water’s Western Treatment Plant (WTP) in Werribee, Victoria, Australia [22], processes raw sewage in treatment lagoons approximately 450 m × 200 m, that are covered with 2 mm thick high-density polyethylene (HDPE) sheets, refer to Figure 1. The covers enable harvesting of the biogas that is produced during anaerobic digestion and is used for generating electricity surplus to the operational requirements of the plant. However, the progressive buildup of semi-solid or solid mass, called scum, which originates from fats, oil, floating solids, buoyed sludge, and other fibrous material, can result in the formation of large mounds commonly referred to as scumbergs. The scumberg can compromise the HDPE floating covers, affecting the structural integrity of the asset, and obstruct the biogas pathway, which consequently diminishes economic returns. Recently, UAV photogrammetry [23,24,25] has been deployed to construct digital elevation models (DEM) and orthomosaic images of the floating cover, enabling monitoring of the asset as well as scum evolution.

Melbourne Water is proactively exploring cutting-edge strategies to augment their capabilities in managing WTP lagoons, aligning with the advancements of Industry 4.0. In particular, they are seeking innovative tools that provide quantitative metrics, including uncertainty, for forecasting performance, decision making, and risk management. The anaerobic lagoon is a highly complex system, the optimisation of which involves diverse multidisciplinary processes, including structural integrity monitoring, biogas extraction, chemical reaction balancing, and controlling inlet flows into the anaerobic sewage. Currently, Melbourne Water employs a supervisory control and data acquisition system for remote control and real-time monitoring of devices at WTPs. These devices include numerous valves and pumps for gas extraction, gas and inlet flow sensor meters, and controls for flow inlets and weather stations. Additionally, water quality monitoring involves testing water samples from the lagoon to measure key chemical parameters, aiming to maintain the process of anaerobic digestion. Furthermore, UAV imagery and manual inspections are also conducted to assess the elevation of the floating cover and the characteristics of the scum in the lagoon. However, due to this complexity, analytical modelling of this system is very challenging, and accurate scaling down for laboratory simulation is not reliable, thereby limiting prognostic capabilities. Nonetheless, WTPs are data-rich, and with the availability of new inspection data (e.g., remote imagery), ML modelling becomes a feasible and attractive practical option for SHM. In particular, the inclusion of data relating to scum characteristics underscores the interconnectedness of operational efficiency and structural resilience of WTP assets. This suggests that predictive models focused on biogas performance can also contribute valuable data for SHM. Therefore, this enables real-time monitoring and the development of a diagnostic-prognostic data-driven model based on actual operational measurements of the sewage processing plant.

Previous work developed safe and rapid non-contact techniques for acquiring information on the deformation and solid scum accumulation under the floating cover [23,24,26]. More recent work aims to leverage these asset-based diagnostics to achieve the performance targets of the cover, such as the biogas collection rate. The goal is to use these data to make informed decisions that ensure the cover is meeting its performance criteria while maintaining its structural integrity. Of particular interest in this research is ML, trained to predict biogas collection rates utilising both operational and inspection sensor data from a WTP, as well as the associated data processing and analysis. Previous studies have demonstrated that RNNs can achieve both deterministic and probabilistic biogas predictions based on operational and environmental conditions over a period of 2 years [27,28]. In this paper, the research is extended by introducing a novel approach to handling asymmetric probabilistic distributions in assimilating the real world, a capability not extensively explored in prior studies. This research further enhances reliable prediction methodologies by integrating a model calibration technique that ensures predictions closely mirror true values. Moreover, the study incorporates and preprocesses data from diverse disciplines, including inspection and chemical parameters, as well as image-based data (DEM and orthomosaics), to model complex assets with multiple dependencies. This interdisciplinary approach, along with our focus on complex probabilistic modelling, distinguishes our work from previous efforts and addresses a critical gap in the application of ML for SHM and asset management. The paper’s presentation is organised as follows: It begins with an overview of the neural network architecture, followed by the preprocessing of WTP historical datasets for ML model training. Subsequently, the paper presents a novel advancement in monitoring systems by incorporating a tailored deep learning BNN architecture to probabilistically predict biogas. Additionally, the significance of different hyperparameter configurations is investigated and discussed. Furthermore, with the hyperparameter-optimised model, its uncertainty is calibrated to quantify various sources of uncertainty. This approach not only showcases the applications of data analytics techniques but also enhances the utility of sensor data in the environmental and energy sectors in readiness of ML modelling for infrastructure management and SHM. The overarching objective of our research project is to transform the assets at WTPs into a smart structure that autonomously regulates both its anaerobic reactor performance and structural integrity, aligning with the principles of the digital twin concept.

## 2. Neural Network Architecture

### 2.1. Recurrent Neural Networks and LSTM

Recurrent neural networks (RNNs) are a type of neural network architecture that is suited for handling sequential data, such as time series data, by using a hidden state that updates with each step, integrating past and present information. However, in practice, they struggle with long-term dependencies due to gradient vanishing. More advanced RNN architectures have been developed, such as long short-term memory (LSTM) networks, that outperform traditional RNNs in capturing dependency. LSTM cells differ from standard recurrent cells in two primary parts. Firstly, the cell state is divided into long-term state $c_{t}$ and short-term state $h_{t}$, and secondly, three control gates (the forget gate, the input gate, and the output gate) are introduced to regulate the flow of information through the cell state. The LSTM operates as follows:

The forget gate $f_{t}$, controls the amount of information to be removed from the previous long-term state $c_{t-1}$ via the following formula:

(1) $$f_{t}=\sigma \left(W_{x,f}x_{t}+W_{h,f}h_{t-1}+b_{f}\right)$$

where σ is the sigmoid function, $x_{t}$ is the input at time step t, and W and b are the weights and bias vectors, respectively, of the corresponding gate and layer.

Secondly, the LSTM cell determines what new information is to be stored in its cell state. The input gate $i_{t}$ regulates the flow of new information from the current output gate $g_{t}$ into the current long-term state $c_{t}$, while the output gate $o_{t}$ controls the formation of the current short-term state $h_{t}$ using the information from the current long-term state $c_{t}$. Both input gate $i_{t}$ and output gate $o_{t}$ can be computed similarly as in Equation (1), using Equations (2) and (3), respectfully:

(2) $$i_{t}=\sigma \left(W_{x,i}x_{t}+W_{h,i}h_{t-1}+b_{i}\right)$$

(3) $$o_{t}=\sigma \left(W_{x,o}x_{t}+W_{h,o}h_{t-1}+b_{o}\right)$$

Next, a vector of new candidate memory content $g_{t}$ is created that may be added to the state, which is given by:

(4) $$g_{t}=\phi \left(W_{x,g}x_{t}+W_{h,g}h_{t-1}+b_{g}\right)$$

where ϕ is the hyperbolic tangent (tanh) function.

In the LSTM cell, the subsequent step involves updating the previous cell state $c_{t-1}$ to the new cell state $c_{t}$. This is achieved by multiplying the old state by $f_{t}$, which removes the information deemed irrelevant by the LSTM cell. Then, only a portion of the output $g_{t}$ is transferred into the current states $c_{t}$ and $h_{t}$. The value of cell state $c_{t}$ is then passed through the tanh filter and multiplied by the output of the output gate layer, resulting in the output of the LSTM cell, which is essentially $h_{t}$. Finally, the equations governing the cell output for the long-term state and the short-term state can be obtained as, respectively:

(5) $$c_{t}=f_{t}*c_{t-1}+i_{t}*g_{t}$$

(6) $$h_{t}=o_{t}*\phi (c_{t})$$

where ∗ is element-wise multiplication.

### 2.2. Bidirectional Long Short-Term Memory

The bidirectional LSTM (BiLSTM) structure is designed to incorporate information that flows both forwards and backwards simultaneously. This is accomplished by utilising two separate LSTMs, which merge data from both preceding and succeeding sequences. Furthermore, the two LSTM neural network parameters in BiLSTM networks are independent but share the same inputs. This approach enhances context comprehension by uncovering system variations and highly nonlinear patterns, while also smoothing predictions for a more accurate representation of the input sequence [29]. The governing equations in the backwards path correspond to the previous Equations (1)–(6):

(7) $${\overset{⃐}{f}}_{t}=\sigma \left({\overset{⃐}{W}}_{x,f}x_{t}+{\overset{⃐}{W}}_{h,f}{\overset{⃐}{h}}_{t+1}+{\overset{⃐}{b}}_{f}\right)\;{\overset{⃐}{i}}_{t}=\sigma \left({\overset{⃐}{W}}_{x,i}x_{t}+{\overset{⃐}{W}}_{h,i}{\overset{⃐}{h}}_{t+1}+{\overset{⃐}{b}}_{i}\right)\;{\overset{⃐}{o}}_{t}=\sigma \left({\overset{⃐}{W}}_{x,o}x_{t}+{\overset{⃐}{W}}_{h,o}{\overset{⃐}{h}}_{t+1}+{\overset{⃐}{b}}_{o}\right)\;{\overset{⃐}{g}}_{t}=\phi \left({\overset{⃐}{W}}_{x,g}x_{t}+{\overset{⃐}{W}}_{h,g}{\overset{⃐}{h}}_{t+1}+{\overset{⃐}{b}}_{g}\right)\;{\overset{⃐}{c}}_{t}={\overset{⃐}{f}}_{t}*{\overset{⃐}{c}}_{t+1}+{\overset{⃐}{i}}_{t}*{\overset{⃐}{g}}_{t}\;{\overset{⃐}{h}}_{t}={\overset{⃐}{o}}_{t}*\phi ({\overset{⃐}{c}}_{t})$$

where the leftward arrow accent indicates the backward direction path.

At each time step t, the forward LSTM generates the hidden state ${\overset{⃑}{h}}_{t}$ based on the previous hidden ${\overset{⃑}{h}}_{t-1}$ and the input vector $x_{t}$, while the backward LSTM generates the hidden state ${\overset{⃐}{h}}_{t}$ based on the future hidden state ${\overset{⃐}{h}}_{t+1}$ and the input vector $x_{t}$. The final hidden state of the BiLSTM model is then formed by concatenating the hidden vectors of both directions. The final output $h_{t}$ of the BiLSTM model at step t is given by:

(8) $$h_{t}=[{\overset{⃑}{h}}_{t},{\overset{⃐}{h}}_{t}]$$

where from hereafter, the forward direction hidden state from Equation (6) is replaced and denoted as ${\overset{⃑}{h}}_{t}$.

### 2.3. Attention Mechanism

Although LSTM and BiLSTM are proficient in handling long-term dependencies, they can face challenges with extremely long sequences due to their limited ability to compress information [30]. The attention mechanism addresses this by focusing on specific sections of arbitrarily long input sequences using attention weights, rather than considering the entire input equally. Earlier studies [2] show that LSTMs with attention outperform traditional LSTMs and other ML algorithms.

The attention mechanism can be integrated with an BiLSTM layer, enabling the allocation of distinct weights to components of the hidden state vector during prediction. Unlike the traditional BiLSTM layer, where the final hidden state vector $h_{T}$, where T is the sequence length, solely encodes the sequence information, the attention mechanism leverages all hidden states ($h_{1},h_{2},\;\ldots ,\;h_{T}$) to construct the context vector $c_{t}$. An attention score $s_{z}$ is computed for each hidden state $h_{z}$ based on a similarity measure between the current BiLSTM hidden state and the corresponding element of $c_{t}$. The score is then normalised using a SoftMax function to obtain the attention weights $a_{z}$, which are parameterised by a single-layer feed-forward neural network. These weights are then applied to compute a weighted sum of the BiLSTM hidden states, forming the context vector $c_{t}$. This essentially gives importance to different temporal information by emphasising the contribution of hidden states that are most relevant to the current time step t.

(9) $$c_{t}=\sum_{z=1}^{T}a_{z}h_{z}\;a_{z}=SoftMax\left(s_{z}\right)=e^{s_{z}}/\sum_{\xi =1}^{T}e^{s_{\xi }}\;s_{z}=v_{a}^{⊺}\phi \left(W_{1,a}h_{z}+{W_{2,a}h_{t}+b}_{a}\right)$$

where $v_{a}$, $W_{a}$, and $b_{a}$ and are learnable weights and biases for the attention layer, and $h_{z}$ and $h_{t}$ are the $z^{th}$ and current BiLSTM hidden states, respectively. Finally, $c_{t}$ can then be passed through the next layer in the neural network.

### 2.4. Bayesian Neural Networks for Epistemic Uncertainty

Implementing BNNs is a challenging and computationally intensive task due to the need for exact Bayesian inference, which involves computing the joint probability distribution of weights given to the observed data. To address these challenges, approximate inference techniques, such as variational inference (VI), have been developed. In the VI approach, weights and bias terms are assigned variational distributions instead of fixed values, unlike in deterministic neural networks [9]. However, the training of VI-BNNs can be computationally expensive due to the increased number of parameters.

Alternatively, an elegant approach for converting a neural network to a Bayesian variant is the Monte Carlo (MC) dropout method [10], which performs approximate inference without changing the entire neural network architecture. Originally, the standard dropout was a widely-used regularisation technique in neural networks, where network neurons are stochastically dropped out with a dropout probability p during model training [31]. The MC dropout method involves conducting multiple stochastic feed-forward passes through the neural network with dropout probability $p_{MC}$ at each layer during the inference phase, which occurs after the model has been trained. This allows for the estimation of both the predictive mean and uncertainty, which are computed as the average and variance of the sampled model outputs, respectively.

Given $y^{*}=f(x^{*})$ is the trained model’s prediction of the new input $x^{*}$, the predictive mean ${\bar{y}}^{*}$ is estimated as the average of H output vectors obtained from H stochastic feed-forwards passes [10]:

(10) $${\bar{y}}^{*}=\frac{1}{H}\sum_{j=1}^{H}{\hat{y}}_{j}^{*}$$

where ${\hat{y}}_{j}^{*}$ is the sample prediction from the $j^{th}$ stochastic forward pass through the neural network with dropout applied.

Similarly, the predictive variance is also estimated as the variance of H output vectors obtained from H stochastic feed-forward passes:

(11) $$Var\left(f\left(x^{*}\right)\right)={\bar{\sigma }}_{E}^{2}=\frac{1}{H}\sum_{j=1}^{H}{\left({\hat{y}}_{j}^{*}-{\bar{y}}^{*}\right)}^{2}$$

This variance, known as model (epistemic) uncertainty and denoted as ${\bar{\sigma }}_{E}^{2}$, emerges from insufficient data.

It is important to perform hyperparameter optimisation and model calibration (discussed in the later section) to ensure that the dropout probabilities p and $p_{MC}$, respectively, are set optimally. Excessive dropout can lead to a deterioration in model learning, resulting in inaccurate predictive means. Conversely, setting the dropout probability too low may cause the model to overfit and yield overly confident predictions, leading to unreliable estimates of predictive variance [14,28].

### 2.5. Mixture Density Networks Using Gaussian Mixture Models for Aleatoric Uncertainty

Aleatoric uncertainty refers to the irreducible noise inherent in data due to measurement errors in sensors or equipment. To estimate this in neural networks, outputs are treated as probability distributions, not single point estimates. The network learns to output both the mean and variance of a probability distribution by employing a maximum likelihood inference approach [32,33], with the variance capturing the aleatoric uncertainty in the training data.

Mixture density networks (MDNs) are an extension of deep learning neural networks that can model complex probability distribution, including asymmetrical and multi-model distributions over continuous variables. MDNs output the parameters of a mixture model, which consists of multiple underlying probability distributions, each being a predefined type of the same family distributions. In general, the probability density for a mixture is defined as follows:

(12) $$p\left(y|x\right)=\sum_{k=1}^{N}\alpha_{k}\left(x\right)D\left(y|\lambda_{1,k}\left(x\right),\lambda_{2,k}\left(x\right),\ldots \right)\;\sum_{k=1}^{N}\alpha_{k}=1,0\leq \alpha_{k}\leq 1$$

where D is the corresponding parametric distribution, λ denotes the parameters of the distribution D, k denotes the index of the corresponding mixture component, N is the number of components in the mixture, and $\alpha_{k}$ is the mixture weights that sums to unity.

Gaussian mixture models (GMMs) are a popular choice for mixture models used in MDNs for modelling arbitrary probability density [2]. In a GMM, the probability density function is modelled as a weighted sum of Gaussian distributions and, hence, the MDN learns the parameters of the GMM, including the mean and standard deviation of each Gaussian component and the weights.

Moreover, in the case where the output distribution layer of a network neural model is a GMM, then:

(13) $$p\left(y|x,\;\theta \right)=\sum_{k=1}^{N}\alpha_{k}\left(x,\theta \right)\;N\left(y|\mu_{k}\left(x,\theta \right),\sigma_{k}^{2}\left(x,\theta \right)\right)\;$$

where $\mu_{k}$ and $\sigma_{k}$ are the conditional mean and standard deviation of the kth Gaussian component, respectively. The neural network outputs a set of values, $a_{k}$, $\mu_{k}$, and $\sigma_{k}$, when given input x, with θ representing the parameters of the network that need to be optimised. In MDNs, N is an additional hyperparameter that can be optimised through optimisation.

Furthermore, the mean of the GMM, $\mu_{GMM}$, is computed as the weighted sum of individual means of the Gaussian component weighted by their mixture weights, as follows:

(14) $$\mu_{GMM}=\sum_{k=1}^{N}\alpha_{k}\;\mu_{k}$$

The mixture variance, $\sigma_{mix}^{2}$, as the weighted sum of the individual variances of the Gaussian component, is weighted by their mixture weights, as follows:

(15) $$\sigma_{mix}^{2}=\sum_{k=1}^{N}\alpha_{k}\;\sigma_{k}^{2}$$

Therefore, the variance of the GMM, $\sigma_{GMM}^{2}$, which is also the aleatoric uncertainty $\sigma_{A}^{2}$, is the sum of the mixture variance and correction term $\mu_{C}$, which accounts for the distance between the mean and the centroid of the GMM, as follows:

(16) $$\sigma_{GMM}^{2}=\sigma_{A}^{2}=\sigma_{mix}^{2}+{\left\|\mu_{GMM}-\mu_{C}\right\|}^{2}$$

where

$$\mu_{C}=\frac{1}{\alpha_{T}}\sum_{k=1}^{N}\alpha_{k}\;\mu_{k},\;\alpha_{T}=\sum_{k=1}^{N}\alpha_{k}$$

### 2.6. Proposed Bayesian Mixture Density Neural Network Architecture

**Figure 2:**
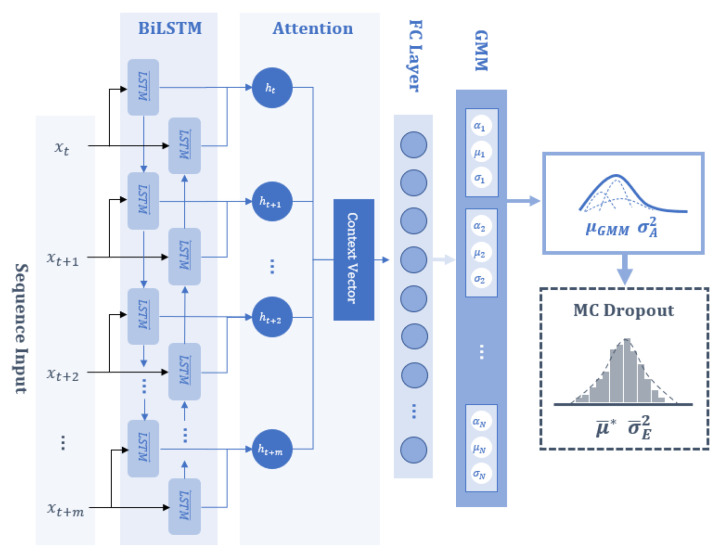
Proposed Bayesian MDN architecture.

The aim of this study is to develop a Bayesian MDN model with an attention-based BiLSTM to predict the biogas collection rate at the next time step and to establish confidence bounds, providing probabilistic estimates of the outcome. The proposed neural network architecture primarily consists of a sequence input layer, followed by a BiLSTM layer with an attention mechanism, a fully-connected (FC) layer with the tanh activation function, and a mixture density distribution output layer based on the GMM, as shown in Figure 2. Furthermore, dropout layers are included in the BiLSTM, attention, and FC layers. The model summary, indicating the array and output shape of each layer, is shown in Table 1. In describing the proposed model, the following hyperparameters are defined: the input window size (m) in the sequence layer, the number of hidden units (h) in the BiLSTM layer, the number of neurons (n) in the FC layer, the dropout probability (p), and the number (N) of Gaussian components for the mixture density distribution output layer. Referring to Table 1, the input layer utilises previous and current timesteps of input vectors $x_{t},\;x_{t-1},\;\ldots ,\;x_{t-m}$. This configuration ensures that the input shape is an array with dimension m by the number of input variables i, which is determined in Section 3. The BiLSTM layer’s output feature size is 2 h for each time step m, and the attention layer summarises the sequence into a single vector of length 2 h. The GMM layer comprises an FC layer without activation, which is designed to generate parameters for this mixture model, outputting a vector of size 3 N that represents the mixture parameters ($\mu_{k}$, $\sigma_{k}^{2}$, and $\alpha_{k}$) and finally, the mixture normal distribution, for which outputs of the GMM are $\mu_{GMM}$ and $\sigma_{A}^{2}$.

The time series dataset is partitioned according to an 80–20 time series train–validation sequential split, where the initial 80% of the data are used for training and the remaining 20% are used for validation. The objective loss function for estimating the parameters of the Gaussian mixture distributions is the negative log-likelihood (NLL) [33]. To mitigate overfitting, an early stopping callback function with a patience value of 100 epochs was implemented. This stops training if there is no improvement in the validation NLL and restores the best model weights based on validation NLL loss. Model optimisation was performed with Adam [34], with a learning rate of 0.001, using the exponential decay rate for the first- and second-moment estimates $\beta_{1}$ and $\beta_{2}$, with values of 0.9 and 0.999, respectively, and a minibatch size of 64.

Neural networks have various hyperparameters, i.e., the number of neurons, number of hidden layers, learning rates and type, etc., which are optimised to obtain the most accurate hyperparameter configuration for any given model. However, the model parameters (weights and biases) are generally discovered by backpropagation optimisation, while hyperparameters cannot be discovered through this training process. Instead, techniques such as exhaustive methods, including grid search and random search, are used to identify the optimal hyperparameter configuration [11]. In this study, the grid search method was performed to analyse and optimise the hyperparameters, generating a total of 3840 candidates from a specified subset of the hyperparameter space, as detailed in Table 2. The tuned hyperparameters included the previous defined hyperparameters: m, h, n, p, and N.

The hyperparameters were initially analysed statistically to identify any significant differences in their impact on the model’s performance. A nonparametric Friedman test assessed if model performance was significantly affected by varying a single hyperparameter while keeping others constant. The null hypothesis stated no significant difference in performance among the hyperparameter settings and, therefore, any observed differences were due to random variability. Subsequentially, a post-hoc Wilcoxon signed-rank test determined the statistical significance of performance between two models with different values of one varying hyperparameter (with the remaining hyperparameters were held constant). Additionally, a Bonferroni correction was applied to the Wilcoxon test by dividing the significance level of 0.05 by the number of pairwise comparisons to control the likelihood of family-wise (probability of at least 1 false positive) error rate for multiple comparisons.

After hyperparameter optimisation, the model with the lowest validation NLL loss was considered the optimal model. This model underwent the MC dropout method with varying values of $p_{MC}$ to predict biogas performance and associated uncertainty intervals. MC dropout was applied to all layers during the testing phase and $\mu_{GMM}$ was sampled to determine the sampled mean ${\bar{\mu }}^{*}$ and variance ${\bar{\sigma }}_{E}^{2}$ (epistemic uncertainty). Preliminary validation confirmed that H≥100 feed-forward passes were sufficient for a relative percentage change of less than 5% in both predictive mean and standard deviation. Finally, assuming that aleatoric and epistemic uncertainties were independent, their combination yielded the total variance. Subsequently, this total uncertainty and their coefficients of variance (CVs) were then analysed [33].

### 2.7. Model Calibration

The calibration of the optimised model was evaluated using a post-hoc calibration method based on the expected calibration error (ECE). ECE measures the discrepancy between the model’s expected confidence and its empirical accuracy [15,16,17,18], thereby assessing the extent to which the model’s predictive uncertainty corresponds to its accuracy. ECE is calculated by first partitioning a set of predictions into equally spaced bins according to their predicted confidence values. For each bin, the expected confidence is determined as the average of the confidence values. For classification problems, empirical accuracy is calculated as the fraction of correct predictions, while for regression problems, it is calculated as a measure of error between the predicted and actual values of the samples within the bin. ECE is then computed as a weighted average of the absolute difference between the confidence and accuracy across all bins. The general form of ECE can be expressed as follows:

(17) $$ECE=\sum_{b=1}^{B}\frac{\left|B_{b}\right|}{K}\left|acc\left(B_{b}\right)-conf\left(B_{b}\right)\right|$$

where K is the total number of predictions, B is the total number of equally spaced bins, $\left|B_{b}\right|$ is the number of samples in bin b, and $acc(B_{b})$ and $conf(B_{b})$ are the empirical accuracy and expected confidence of bin b, respectively. 

In accordance with prior works [17,18,19], the ECE for regression approach was applied to our study application. Given a test dataset of K predictions, where the predictive mean and model uncertainty ${\bar{y}}_{l}^{*}$ and ${\hat{\sigma }}_{E_{l}}$ are extracted via MC dropout for the $l^{th}$ sample of the test dataset, the predicted uncertainty range was partitioned into B equally spaced bins. Each sample was then assigned to the corresponding bin based on its uncertainty value. For each bin b, the empirical accuracy was computed as the average absolute difference between the predictive mean and the true value for the samples assigned to bin b. Similarly, the expected confidence was calculated as the mean predictive uncertainty of the model for the samples in bin b. Therefore, the empirical accuracy and expected variances for bin b are given by:

(18) $$acc\left(B_{b}\right)=\frac{1}{\left|B_{b}\right|}\sum_{l\;\in {\;B}_{b}}|y_{l}-{\bar{y}}_{l}^{*}|\;conf\left(B_{b}\right)=\frac{1}{\left|B_{b}\right|}\sum_{l\;\in {\;B}_{b}}{\hat{\sigma }}_{E_{l}}$$

With the accuracy and confidence defined using Equation (18), ECE was determined using Equation (17). A lower ECE signifies better calibration, indicating more accurate and reliable uncertainty estimates from the model. Conversely, a high ECE indicates poor calibration, suggesting that the predicted uncertainties are not good estimates of the true uncertainties. In this study, three different B values, 10, 50 and 100, were used to evaluate the model uncertainty of the optimal model with varying $p_{MC}$, ranging from 0.01 to 0.80.

## 3. Method

### 3.1. Data Preparation

A historical time series dataset, consisting of measurements from pump and flow sensors as well as chemical and weather measurements of a real-world 55E anaerobic lagoon at a WTP, was used in this study, refer to Table 3. The compilation of the data was undertaken by WTP process engineers, expert technicians, and researchers in their respective fields. The dataset comprised 24 operational and environmental variables recorded daily from 1 January 2012, to 26 August 2019, resulting in a total of 2795 readings. The variables included average, maximum, and minimum biogas collection rates (g), Inlet A ($I^{A}$) and Inlet B ($I^{B}$) readings, ambient temperature ($T^{A}$), solar exposure (S), and rainfall (R). Additionally, the dataset also comprised six chemical field and laboratory variables, such as pH level (pH), alkalinity (ALK), biochemical oxygen demand (BOD), chemical oxygen demand (COD), filtered chemical oxygen demand (FCOD), and volatile fatty acids (VFA). It is important to note that the data were irregular, i.e., COD had non-uniform readings taken every 0.65–14.04 days with a median time step of 1.01 days, containing 1688 data points.

### 3.2. Inspection Parameters

#### Scum Representative Variable Using Scum Depth Surveys and Digital Elevation Models

In addition to the aforementioned variables, the study also incorporated the inspection parameters, which included digital elevation models (DEMs) and their associated orthomosaics and scum depth surveys. A total of nine scum depth surveys were conducted approximately every 5 months to 1 year from August 2014 to January 2018. These surveys involved manually measuring the scum depth at 32 different portholes on the cover. Specifically, authorised field personnel inserted a long, rigid rod into the access ports, and the scum depth was recorded when encountering a transition in resistance from solid to liquid sewage, with a tolerance of 100 mm. Additionally, six DEMs and orthomosaics of the floating cover asset in 2019 (January to September) were utilised as variables for this study. To address the irregularity of the limited available raw inspection data, assumptions were made to regularise and merge these scum survey data and DEMs into a single cohesive dataset.

The process of combining two datasets into a single variable involves using a relationship between two variables to derive a single variable for each data point. Previous work by Wong et al. [23] showed a linear association between scum depth and DEM elevation, which accounted for 77% of the variability in scum depth, with a linear gradient of 3.55 at a different WTP anaerobic lagoon. The relationship of the elevation of the 55E floating cover was first validated using by performing a Pearson’s correlation test using the WTP laser measurements and scum depth surveys recorded in January 2018. The correlation test indicated a strong linear relationship, with a coefficient of 0.83 and a strong statistical significance of *p*-*value* < 10^−7^. Subsequently, a robust linear regression using the bisquare method was conducted to determine the linear model. To ensure more reliable and unbiased estimates of the coefficients and error measurements, 13 zero readings were excluded from the 32 laser measurements. According to the regression model, approximately 65.8% of the observed variability in the scum depth could be explained by the model, with a root-mean-square error of 394.5 mm. The findings indicated that for each one-unit rise in elevation, the scum depth increased by a factor of 3.59 with a positive offset of 27.78 mm. This linear relationship allowed for the combination of both DEM and scum survey datasets into a single variable representing the scum depth. This was accomplished by considering the elevations within the vicinity of the 32 portholes in the DEM. However, it is important to note that the elevations are susceptible to noise, often requiring intensive preprocessing steps to filter out unwanted features prior to analysis [35]. In this context, undesirable artefacts such as trapped rainwater and dirt introduce significant variations in displacement readings, distorting the accuracy of elevation measurements for analysis [23,25].

In our previous work [25], an improved k-means with a centroid initialisation technique clustering method was introduced to filter unwanted artefacts, such as water features, debris/dirt, and man-made objects, in WTP anaerobic lagoons. The premise was that the artefact and noise clusters could be rapidly identified through a visual examination and hence removed by excluding these clusters. It was shown that using the k-means filtering method to remove water features achieved a relative error of 3.8% with respect to on-site laser measurements, compared to a relative error of at least 17.2% when using classical methods (such as median filters). This study incorporates the k-means filtering method and the procedure is as follows:

- The DEMs and their associated orthomosaics are stacked as a 4D array to enable the algorithm to cluster features into distinct groups.
- The Calinski–Harabasz (CH) criterion, which measures the between-cluster variance and within-cluster variance, is then employed to determine the optimal k groups. The optimal k groups correspond to the highest CH index, by inspecting k from 0 to 10.
- The algorithm proceeds with the optimal k groupings and the resulting clusters with features not associated with the membrane cover are considered artefacts. Thereby, the remaining clusters are then merged to provide a filtered DEM.

Further details on the k-means filtering procedure can be found in [25].

**Figure 3:**
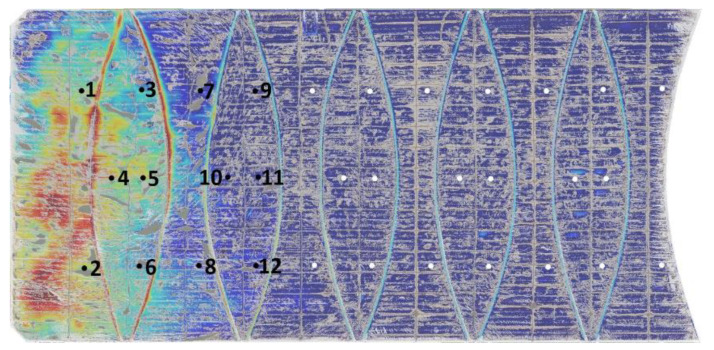
K-means filtered elevation profile of DEM overlayed on its associated orthomosaic with labelled portholes.

Porthole elevation measurements from the filtered DEM were derived by calculating the median displacement within a 2 m-by-2 m localised region centred at each porthole location, refer to Figure 3. This process was repeated for all DEMs captured at various times. Scum depth measurements at 20 porthole locations were found to be essentially zero [24], and thus deemed redundant. Consequently, measurements at these porthole locations were excluded from the study and, therefore, the analysis focused solely on the scum depth measurements from the remaining 12 porthole locations, as indicated in Figure 3. Consequently, the scum depth measurements obtained from both the scum survey and the filtered DEMs were merged. In this context, $P_{q}$ represents the scum depth value at the q-th porthole location, and the statistics of each $P_{q}$ as shown in Table 4.

### 3.3. Reduction of Data Dimensionality

In practical applications, the reduction of inputs by eliminating redundant variables is highly advantageous. This approach helps to mitigate risks associated with overfitting and multicollinearity and expedites the training process by simplifying the model. Our previous investigations [27,28] served as the basis for employing a feature selection technique based on correlation for input dimensionality reduction. In our previous work on data preprocessing, it was demonstrated that reducing the number of input variables by removing those that are highly correlated enhances model accuracy. This was achieved by employing a thresholding approach in pairwise Spearman’s Rank correlation analysis. Variables exhibiting coefficient values greater than 0.75 were identified as highly correlated and subsequently eliminated [27]. In this study, considering a total of 36 variables, further exploration of this threshold value using an incremental step size of 0.05 revealed that the number of variables reduced to eight remained constant between threshold values of 0.35 and 0.8, and beyond a threshold value of 0.8, more than 25 variables were retained. Therefore, the work maintained a threshold of 0.75 for correlation-based dimensionality reduction to effectively balance model simplification with the retention of significant variables.

**Figure 4:**
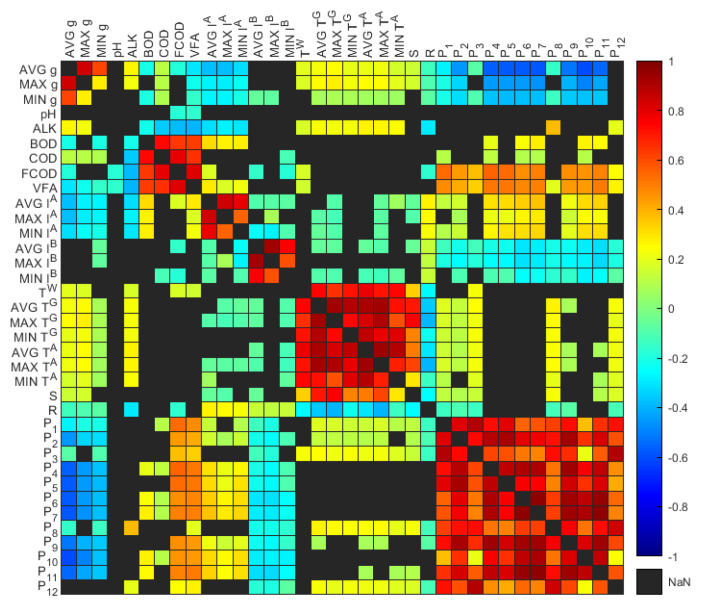
Heatmap of Spearman’s piecewise correlation matrix on the variables showing *p*-value < 0.05.

With the highly correlated variables identified, the rationale behind the retention and removal of variables is based on their representativeness, quality, and statistical properties. As shown in Figure 4, the average, maximum, and minimum variables of biogas exhibited positive correlations, and this correlation pattern was also observed for Inlet A, Inlet B, and temperature variables. The average values of these variables were retained because they provided a more comprehensive overview, reflecting general conditions rather than extreme values, refer to Table 3. Furthermore, the temperature readings and S demonstrated a strong positive correlation, thereby leading to the removal of S. The scum depth readings at each porthole were also highly positively correlated. However, $P_{2}$ and $P_{8}$ were retained based on their location, which is significant for the distribution of the scum geometry and to preserve spatial information. The chemical and organic variables (BOD, COD, FCOD, and VFA)) exhibited strong positive correlations. However, the variables with more than 60% missing data, specifically pH, ALK,BOD,FCOD, and VFA, were not considered and thus removed. Therefore, COD was retained as the more representative chemical variable due to its higher data quality (similar time step and higher data portion) compared to other chemical variables. Consequently, 28 highly correlated variables were eliminated, reducing the total number of representative input variables x to eight unique variables. These included the average values of biogas collection, Inlet A, Inlet B, and ambient temperature, hereafter denoted as g, $I^{A}$, $I^{B}$, and $T^{A}$, respectively, along with COD, R, $P_{2}$ and $P_{8}$.

**Figure 5:**
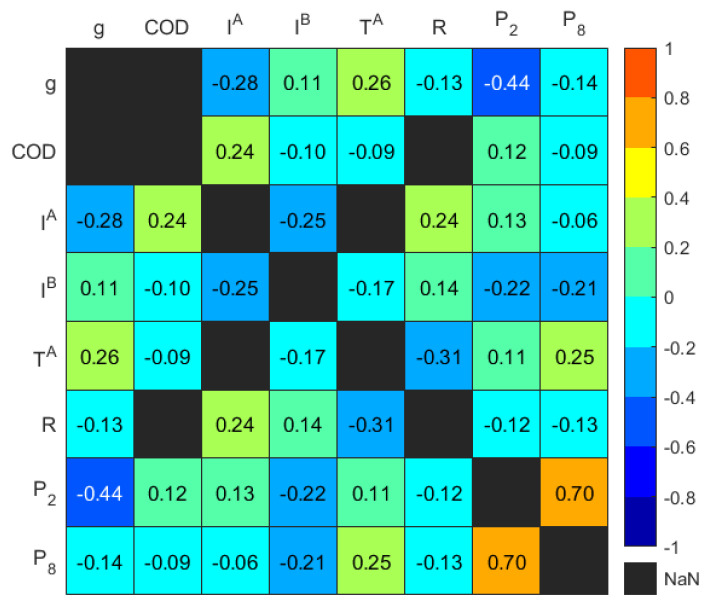
Heatmap of Spearman’s piecewise correlation matrix on the representative input variables showing *p*-value < 0.05.

The correlation coefficients of the representative input variables that had a high level of statistical significance are shown in Figure 5. It is shown that there was a moderate negative correlation between $I^{A}$ and g, as well as between $P_{2}$ and g, with coefficients of −0.28 and −0.44, respectively. Additionally, a moderate positive correlation was found between $T^{A}$ and g, with a coefficient of 0.26. These variables were considered to exert a primary influence, displaying a monotonic-like relationship with g. However, variables with weaker correlations, such as $I^{B}$, were anticipated to exhibit a nonlinear relationship with g, as suggested by previous investigations [28].

### 3.4. Resampling of Irregular Representative Variables

Data regularity is crucial for effectively training a model, as irregular data with biased patterns can introduce spurious relationships between the inputs and outputs, negatively impacting the model’s performance. Several techniques can be employed to address irregular data for model training, such as resampling and interpolation. These techniques can be selectively applied to specific variables based on their unique data characteristics.

**Figure 6:**
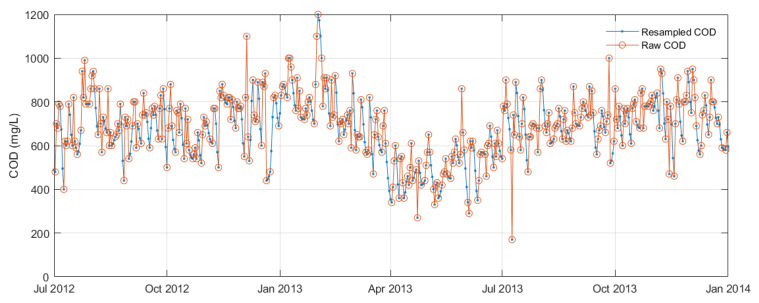
Illustration of resampled and raw COD variables between July 2012 and January 2014, depicting the irregular dataset before data preprocessing.

In this study, the missing rainfall data, which constituted only a small portion of the overall dataset, were replaced with the average value of the preceding and next-day values. To address the irregular time step of the COD variable, a Lomb–Scargle periodogram was initially constructed to confirm the existence of a 7-day cyclic trend. As this trend was relatively longer than the time step, data regularisation was performed using interpolation. For this purpose, a piecewise cubic Hermite interpolation polynomial (PCHIP) with a daily time step was employed, which helped to prevent overshooting and preserve monotonicity, refer to Figure 6.

**Figure 7:**
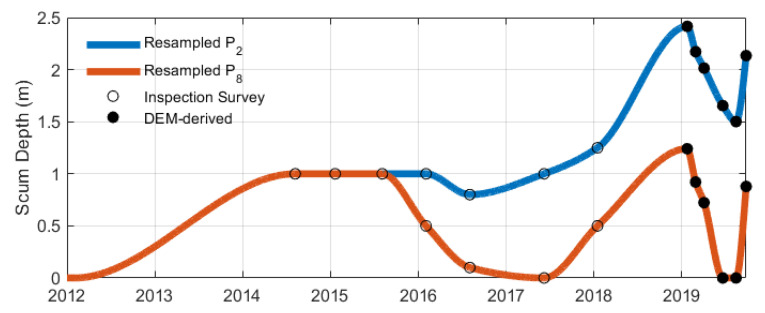
Resampled portholes data $P_{2}$ and $P_{8}$ interpolated using PCHIP, based on inspection surveys and DEM-derived scum depth measurements.

Consequently, the scum depth measurements $P_{2}$ and $P_{8}$ were resampled for model training. Due to the gradual accumulation of scum over an extended period (ranging several months to years) [36], interpolation was considered appropriate for resampling the scum depth. Using a similar interpolation approach as with the COD variable, scum depth measurements were interpolated daily using PCHIP, ensuring the preservation of monotonicity and preventing overshooting, as illustrated in Figure 7.

It was revealed that resampling and preprocessing of the variables COD and R had a negligible effect on their statistical properties, refer to Table 5. In particular, the mean value of the resampled COD showed a difference of 0.7% and its standard deviation showed a difference of 2.1% relative to the unprocessed COD. This indicated accurate interpolation of data in capturing COD’s periodic trend. Furthermore, the interpolated $P_{2}$ and $P_{8}$ exhibited significant differences in mean values, with changes of 26.1% and 12.3% relative to the unprocessed $P_{2}$ and $P_{8}$, respectively. However, this was an expected outcome due to the nonlinear positive trend observed in the scum depth values. Thereby, these eight representative variables, each with 2795 daily measurements, were considered for model training.

Furthermore, our previous work [27] demonstrated that data standardisation, which involved rescaling the data to have a mean of zero and a standard deviation of one, led to a reduction in the mean square error of approximately 15% compared to data normalisation, and it was approximately twice as fast to train, requiring half the number of epochs to achieve an optimal model based on training and validation errors. Hence, in this study, the representative variables underwent data standardisation. This ensured similar scales and distributions among variables, thereby enhancing the neural network’s ability to identify patterns in the dataset, reducing bias towards variables with larger scales, and improving outlier handling [27,37].

## 4. Results

### 4.1. Effects of Hyperparameters

Based on the optimisation of hyperparameters, the optimal model consisted of an input window size of 7, a dropout probability of 0.01, three Gaussian components in the mixture distribution output layer, 12 neurons in the FC layer, and two hidden units in the BiLSTM layer. The optimal model achieved an NLL of 0.074, which was approximately 9 times more accurate than the average model performance (NLL = 0.753) and approximately 22 times that of the worst model (NLL = 1.677).

The Friedman test showed significant differences in model performance across all hyperparameters, refer to Table 6. Notably, the input window size and the number of hidden units (m and h) displayed strong statistical significance in performance differences, suggesting their significant impact on the model’s overall performance.

**Figure 8:**
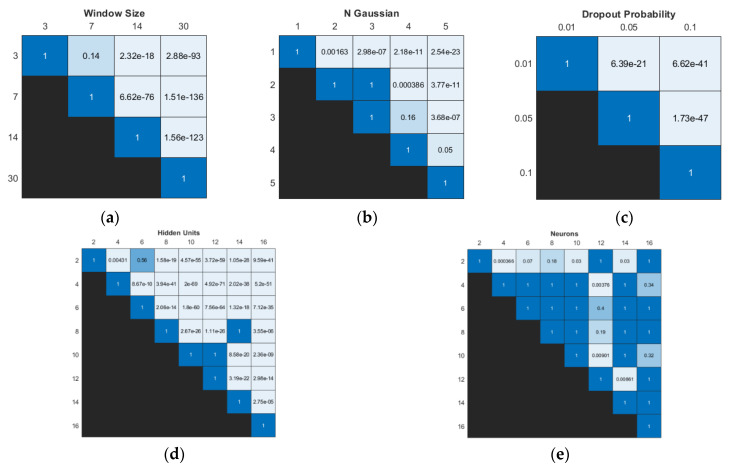
Significance results obtained using Wilcoxon signed-rank test with Bonferroni correction of different values within the same type of hyperparameter: (**a**) input window size, (**b**) number of Gaussian components, (**c**) dropout probability, (**d**) number of hidden units in BiLSTM layer, and (**e**) number of neurons in FC layer.

The Wilcoxon signed-rank test revealed significant differences in model performance for various dropout probability settings, refer to Figure 8. However, for the input window size, no significant differences were observed between the 3- and 7-day models. In terms of the number of hidden units, three pairwise comparisons (2 vs. 6, 8 vs. 14, and 10 vs. 12) showed no significant differences in performance, refer to Figure 8d. Most pairwise comparisons of the number of neurons showed no significant differences. Pairwise comparisons involving 2 and 12 neurons demonstrated the highest number of pairs with some significant differences in model performances.

In Table 7, the results present the average and standard deviation performance of models for a fixed single hyperparameter, along with the proportion of these models that ranked within the top 10% (top performing) and bottom 10% (worst performing) of all models.

The model’s performance was significantly influenced by the dropout probability, with the best average performance observed for p=0.01. However, this value exhibited the highest variance among all dropout probabilities. Notably, approximately 56.8% of models with p=0.01 ranked among the worst performing models. An increase in the number of Gaussian components in the output layer improved the average model performance. The analysis indicated that most top performing models had m≥3. On average, the model performed best with an input window size of 7. By contrast, larger window sizes of 14 and 30 days negatively affected model performance, which indicated overfitting, as a greater number of parameters increases the model’s capacity to assimilate noise and granular details from the training data, thereby resulting in model degradation. It was observed that 75.2% of the worst performing models had larger window sizes of 14 and 30 days, while window sizes of 3 and 7 days were associated with 86.4% of the top performing models.

The best average performance of the model was achieved with 4 hidden units in the BiLSTM layer. Significantly, 30.5% of models with h = 4 were top-performing models, while only 0.5% were among the worst performing models. Additionally, 60.1% of top-performing models had h values of 2 or 4, whereas approximately 85.9% of the worst performing models have more than h > 8.

### 4.2. Epistemic Uncertainty via MC Dropout and Calibration

**Figure 9:**
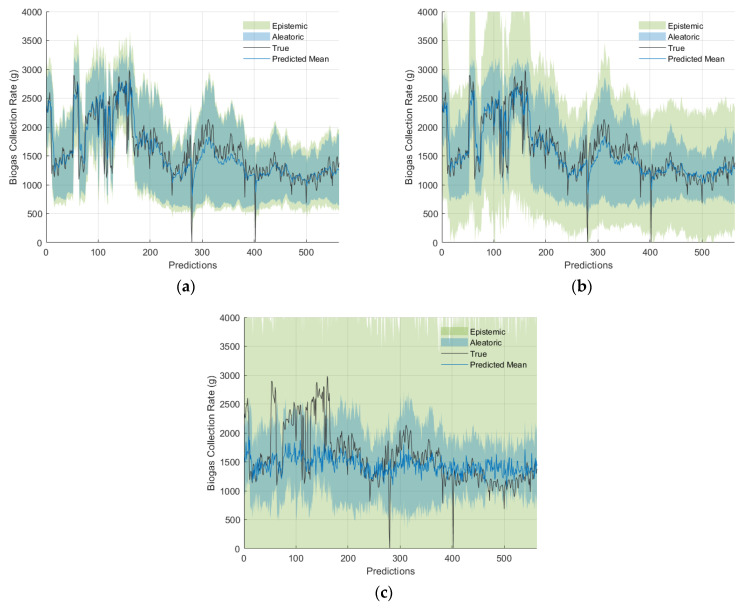
Biogas collection rate predictions and their 95% confidence intervals showing both aleatoric and epistemic uncertainties for different $p_{MC}$ values: (**a**) 0.01, (**b**) 0.20, and (**c**) 0.80.

Figure 9 illustrates examples of biogas collection rate prediction on the validation set for various values of $p_{MC}$, along with their corresponding epistemic and aleatoric uncertainties. It is shown that excessive MC dropout during inference led to an overestimation of uncertainty, which resulted in overly cautious predictions. On the other hand, low MC dropout resulted in an underestimation of uncertainty, leading to potentially overconfident predictions.

**Figure 10:**
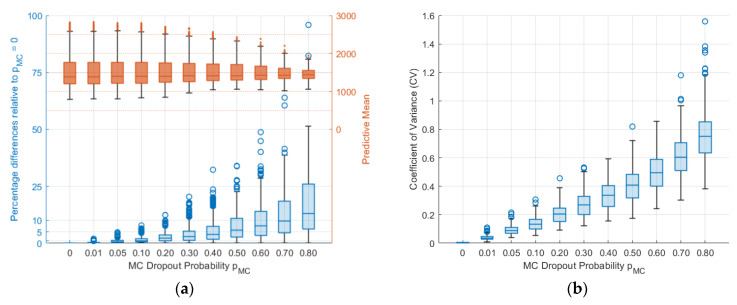
(**a**) Distributions of sampled predictive mean values and their percentage difference relative to those with $p_{MC}=0$; (**b**) CV for different $p_{MC}$ values.

To analyse the differences in predictive means, various $p_{MC}$ values were compared relative to no MC dropout ($p_{MC}=0$). As shown in Figure 9, the relative percentage difference of the predictive mean increased as $p_{MC}$ increased. For $p_{MC}\leq 0.10,$ there was a relative percentage difference of less than 4.5%, with the exception of outliers. Significant relative percentage differences were observed for $p_{MC}=0.70$ and 0.80, with median differences of 9.62% and 12.94%, respectively, and quartile ranges from 4.58% to 18.54% and from 6.1% to 26.1%, respectively. Furthermore, a decrease in the variation of the predictive mean, indicating over-regularisation, is seen in Figure 9 and Figure 10. Figure 10 also demonstrated that as $p_{MC}$ increased, both the CV and its range increased, while the number of outliers decreased as a result of larger CV values.

**Figure 11:**
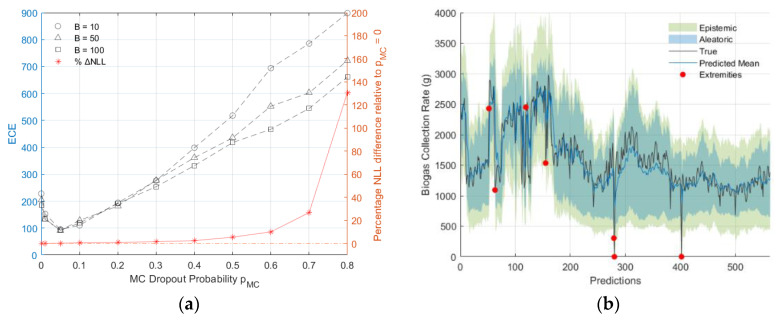
(**a**) ECE values and percentage NLL difference relative to optimised model with $p_{MC}=0$ for different $p_{MC}$ and B size; (**b**) Calibrated and optimised model with 95% confidence interval and indicated extremities.

Referring to Figure 11, among optimised models with different $p_{MC}$, a well-calibrated model with $p_{MC}=0.05$ was indicated by the ECEs from all B values. However, exceeding this $p_{MC}$ value led to increased model miscalibration. Furthermore, it was observed that optimal models with $p_{MC}\leq 0.20$ had negligible effects on model performance, with the difference in NLL relative to those of the optimal model with $p_{MC}=0$ being less than 1%. By contrast, models with $p_{MC}\geq 0.50$ exhibited a relative NLL difference exceeding 5%.

The total uncertainty of the calibrated model was predominantly composed of aleatoric uncertainty, which accounted for 93.7% and was larger than the epistemic uncertainty. The mean values of the aleatoric and epistemic uncertainties were 319.4 and 144.1, respectively. In this model, approximately 1.2% of cases (seven extremities) exceeded the 95% confidence interval. Of these, five cases were below the lower bound (less than the 2.2nd percentile) and the remaining two exceeded the upper bound (more than the 97.7th percentile). Nevertheless, it was shown that the model-identified outliers, which can indicate potential anomalies, could serve as an early warning tool for critical infrastructure.

## 5. Discussion

The optimal input window size corresponded to the 7-day time lag based on the autocorrelation found in the previous study [28]. The findings were consistent with prior work on Bayesian LSTM networks, where aleatoric uncertainty was the least when the optimal dropout probability p=0.01. Additionally, it was also seen that high dropout probabilities can underfit the model and deteriorate the model’s performance in its ability to learn meaningful patterns due to the excessive masking of data [28].

Generally, increasing the number of Gaussian components in a GMM enhances its ability to fit complex distributions, but an excessive number of components can cause overfitting. To further investigate, the best performing model with five Gaussian components revealed that the average ratios of the predicted two smallest mixture weights to the corresponding maximum mixture weight $\frac{a_{k}}{max\left(a_{k}\right)}$ were 3.9% and 7.3%, respectively. Furthermore, the mean values of the smallest mixture weight differed by an average of 3.34% compared to the means corresponding to the remaining mixture weights. This suggested that at least one component in this best performing model captured nuances that did not significantly improve the fit, thereby adding unnecessary complexity and prolonging training time. While this indicated overfitting when exceeding the optimal number of Gaussian components, such evidence was not apparent in the statistical analysis. It is anticipated that incorporating an additional penalty term into the objective function can serve as a remedy to mitigate overfitting. However, this aspect is beyond the scope of the current work.

In this study, it was found that increasing the number of hidden units or neurons did not necessarily improve the model’s accuracy and may adversely affect performance. The results suggested interdependencies among the hyperparameters, as evidenced by the differences between the hyperparameters of the optimal model and those yielding the highest average performances. The sensitivity of certain hyperparameters to model performance is expected to vary depending on the specific application being considered. Thus, it is crucial to optimise hyperparameters and to develop a well-tuned model that can make accurate predictions tailored to the application.

The current work used an exhaustive grid search method with the intent to analyse the hyperparameters. However, cost-effective strategies for tuning hyperparameters in complex models include the random search method [11] and Bayesian optimisation techniques, which consider previously sampled hyperparameters by using a surrogate regression model (i.e., Gaussian process and random forest) [12]. While this study focused on optimising hyperparameters related to the model architecture (i.e., layers, number of neurons, etc.), it is crucial for large, complex neural networks to also consider other hyperparameters related to the learning process of the model, such as optimiser type and their learning parameters. These should be optimised holistically to reduce the training time, particularly for new problem domains, through the transfer of weights or architecture requiring model re-tuning. Furthermore, additional considerations become imperative for larger neural networks, particularly in terms of computational cost, processing and model training time, and power consumption, as well as the intricacies involved in model deployment. However, such challenges are absent in the context of the work and the current proposed neural network architecture but will be considered in future work.

It was observed that MC dropout significantly impacted the uncertainty quantification in the proposed probabilistic neural network. Despite the goodness-of-fit degradation with increasing $p_{MC}$, optimal models with $p_{MC}\leq 0.2$ showed negligible differences in predictive mean, as previously stated. This suggested that these models had a reasonable balance between the calibration of model uncertainty and acceptable model fit, making them valid choices. While $p_{MC}$ can be tuned during hyperparameter optimisation, its higher computational cost due to retraining with different $p_{MC}$ often necessitates post-hoc tuning [19,21]. In this study, it was shown that performing post-hoc calibration is effective since the optimal $p_{MC}$ has a negligible effect on the predictive mean of the model. However, in scenarios where $p_{MC}$ or other hyperparameters significantly influence the predictions, post-hoc calibration and tuning should not be employed. In such cases, incorporating calibration methods into the model’s training process, for instance by modifying the loss function with a penalty term, may offer a more effective and cohesive approach [20].

Considering the specifications of sensors or instrument devices is critical, as their limitations may impact the effectiveness of the learning model. However, this work relied on industry experts for the provision of the dataset, which underwent processing from its initial raw state, including data reformatting and consolidation. It was therefore considered to be of sufficiently high quality for analysis. Although the details of the data preparation process are not specified in this paper, they should be recognised as important, particularly for the ML process. Nevertheless, the primary emphasis of this research is on leveraging data from a diverse range of disciplines, leading to insightful and actionable outcomes using ML in practical applications.

One common challenge in training neural networks, as evident in this study, is the lack of datasets, particularly due to limited real-life inspection. It is important to acknowledge that the current inspection information may not adequately capture the spatial context of the asset, particularly in areas with highly localised elevation or scum depth variations. Additionally, the exclusion of measurements from porthole locations may not be appropriate, particularly in adaptive and reinforcement learning scenarios where models must continuously learn and adapt to new and unseen real-time data. To overcome these limitations, future work will aim to address the dataset scarcity for scum variables by utilising synthetically generated data to train deep learning neural networks in feature extraction. Subsequently, a neural network will be developed in which the portions of its architecture representing the feature extraction network are transferred. Nevertheless, the study demonstrates the effectiveness of the proposed probabilistic neural network in revealing that the inherent data are the primary source of uncertainty, as reflected in larger aleatoric uncertainty estimates. This work establishes a foundational basis for prognostic ML approaches that facilitate SHM and informed decision making in managing WTP anaerobic lagoons.

## 6. Conclusions

In this study, a Bayesian MDN model for anaerobic biogas performance prediction in a WTP was proposed, incorporating uncertainty quantifications from both inherent noise and the model itself. The neural network architecture featured an attention-based mechanism in a BiLSTM layer and a GMM distribution output layer for inherent noise variance. The preprocessing approach simplified complex sensor data and other data types, enabling effective model training and highlighting a critical aspect of developing predictive models SHM. This approach effectively reduced the number of unique variables required for input to eight. The grid search method was employed to determine the optimal model and to investigate the influence of hyperparameters. The results emphasised the significance of hyperparameter optimisation and model uncertainty calibration as essential steps for ensuring accurate predictions. The optimal model achieved an accuracy that surpassed the average model performance by approximately 9 times and significantly outperformed the worst performing model by approximately 22 times. It was also found that the number of hidden units in the BiLSTM layer and the input window size significantly impacted performance, whereas the number of neurons in the FC layer showed no statistically significant difference. The MC dropout method was applied to quantify the model uncertainty, and the post-hoc calibration method using ECE revealed that the model with $p_{MC}=0.05$ exhibited confidence levels that accurately encompassed the true biogas collection rates. Furthermore, the model indicated that the overall uncertainty was largely due to the inherent limitations of the data, revealing the need for additional high-quality data for learning. It is anticipated that, due to the limitations of real-life inspections in describing the spatial context of the asset, further work will be required to address data scarcity. In continuation of our research, the results of this study lay the groundwork for the integration of deep learning in managing and monitoring the entire complex system of anaerobic lagoons and floating covers in WTPs with enhanced diagnostic and prognostic capabilities. Moreover, the methodologies and findings of this study offer significant implications for SHM of infrastructure, promising enhanced capabilities in the field.

## Figures and Tables

**Table 1:** The proposed model summary and array/output shape.

Layers		Array/Output Shape
**Input Shape**		(m, i)
**BiLSTM + Dropout (p)**		(None, m, 2 h)
**Attention Layer + Dropout (p)**		(None, 2 h)
**FC Layer (tanh) + Dropout (p)**		(None, n)
**GMM Layer**	FC Layer (None)	(None, 3 N)
	Mixture Normal Distribution	((None,1), (None,1))

**Table 2:** Hyperparameters for optimisation.

Hyperparameter	Search Grid Range
**Input Window Size m**	[3, 7, 14, 30]
**Dropout Probability p**	[0.01, 0.05, 0.10]
**BiLSTM Layer** **Hidden Units h**	[2–16] increments of 2
**FC Layer** **Neurons n**	[2–16] increments of 2
**GMM Output Layer** **Gaussian Components N**	[1–5]

**Table 3:** Descriptive statistics of the operational and environmental data.

*Variable*	*Units*	*Mean*	*Min*	*Max*	*Standard Deviation*	*Data Portion*	*Time Steps (Days)*	*Median Time Steps* *(Days)*
$g_{avg}$	Nm^3^/hr	1693.6	0	3680.6	843.9	100%	1	1
$g_{max}$	Nm^3^/hr	2197.8	0	5451.0	1033.5	100%	1	1
$g_{min}$	Nm^3^/hr	1098.6	0	3390.0	922.3	100%	1	1
pH	pH Units	6.6	6	7.0	0.1	30.7%	[0.01–20.95]	2.92
ALK	mg/L	378.9	210	580.0	40.8	14.2%	[0.38–20.95]	7
BOD	mg/L	309.6	34	830.0	92.0	32.0%	[0.01–20.95]	1.07
COD	mg/L	690.6	170	1300.0	133.7	60.4%	[0.65–14.04]	1.01
FCOD	mg/L	254.2	69	540.0	62.3	31.9%	[0.37–14.07]	1.02
VFA	mg/L	98.5	10	260.0	54.8	14.2%	[0.38–20.95]	7
$I_{avg}^{A}$	ML/d	199.2	0	428.9	51.3	100%	1	1
$I_{max}^{A}$	ML/d	277.6	0	750.0	79.1	100%	1	1
$I_{min}^{A}$	ML/d	94.4	0	342.0	43.0	100%	1	1
$I_{avg}^{B}$	ML/d	10.7	0	257.1	36.2	100%	1	1
$I_{max}^{B}$	ML/d	27.8	0	595.0	73.8	100%	1	1
$I_{min}^{B}$	ML/d	5.0	0	183.0	18.2	100%	1	1
$T^{w}$	Celsius	20.2	12	25.0	2.0	19.3%	[0.01–20.95]	6.98
$T_{avg}^{G}$	Celsius	14.1	6.867	35.8	5.2	100%	1	1
$T_{max}^{G}$	Celsius	19.8	8.7	51.5	8.4	100%	1	1
$T_{min}^{G}$	Celsius	10.1	0	27.3	3.7	100%	1	1
$T_{avg}^{A}$	Celsius	15.4	4.05	35.1	5.1	100%	1	1
$T_{max}^{A}$	Celsius	20.6	8.1	44.8	6.4	100%	1	1
$T_{min}^{A}$	Celsius	10.3	−2.1	28.9	4.8	100%	1	1
S	MJ/m^2^	14.8	1.3	34.3	8.1	100%	1	1
R	mm	1.2	0	41.0	3.5	99.7%	1	1

**Table 4:** Descriptive statistics of the scum depth, in metres, at 12 portholes.

Porthole	Mean	Min	Max	Standard Deviation
**P_1_**	1.7	0	2.9	0.7
**P_2_**	1.4	0	2.4	0.6
**P_3_**	1.0	0	1.8	0.5
**P_4_**	1.5	0	3.1	1.0
**P_5_**	1.2	0	2.4	0.9
**P_6_**	1.2	0	2.1	0.6
**P_7_**	0.6	0	1.1	0.4
**P_8_**	0.6	0	1.2	0.4
**P_9_**	0.5	0	1.0	0.4
**P_10_**	0.5	0	1.1	0.4
**P_11_**	0.5	0	1.0	0.3
**P_12_**	0.5	0	1.0	0.4

**Table 5:** Descriptive statistics of the representative variables, including those resampled and interpolated.

*Representative Variable*	*Unit*	*Mean*	*Min*	*Max*	*Standard Deviation*
g	$Nm^{3}$/hr	1693.6	0	3680.6	843.9
COD	mg/L	686.2	170	1300.0	130.9
$I^{A}$	ML/d	199.2	0	428.9	51.3
$I^{B}$	ML/d	10.7	0	257.1	36.2
$T^{A}$	Celsius	15.4	4.05	35.1	5.1
R	mm	1.2	0	41.0	3.5
$P_{2}$	m	1	0	2.4	0.6
$P_{8}$	m	0.5	0	1.2	0.4

**Table 6:** Friedman test on the hyperparameters.

	Window Size	Dropout Probability	Hidden Units	Neurons	Gaussian Components
	m	p	h	n	N
***p***-value	1.23 × 10^−251^	1.50 × 10^−37^	7.11 × 10^−212^	1.40 × 10^−5^	5.03 × 10^−22^

**Table 7:** Statistical summary of average model performance for each hyperparameter. Bolded is the best value of the corresponding statistics.

	Average NLL	Standard Deviation NLL	Top 10%	Bottom 10%
Dropout Probability p				
0.01	**0.715**	0.216	30.2%	56.8%
0.05	0.751	0.185	31.5%	31.3%
0.1	0.793	**0.163**	**38.3%**	**12.0%**
Gaussian Components N				
1	0.795	0.198	12.5%	33.9%
2	0.765	0.186	16.9%	23.4%
3	0.751	0.192	21.4%	18.0%
4	0.738	0.195	**24.7%**	21.1%
5	**0.716**	**0.179**	24.5%	**3.6%**
Window Size m				
3	0.700	0.210	**47.9%**	**10.9%**
7	**0.689**	0.186	38.5%	13.8%
14	0.761	0.169	12.0%	26.3%
30	0.860	**0.148**	1.6%	49.0%
Neurons n				
2	**0.728**	0.197	7.6%	16.1%
4	0.770	0.179	15.1%	**8.3%**
6	0.754	0.190	13.8%	14.8%
8	0.758	0.207	**16.4%**	14.3%
10	0.769	0.198	13.3%	12.8%
12	0.734	0.196	12.8%	14.1%
14	0.762	0.196	11.7%	10.7%
16	0.747	**0.168**	9.4%	8.9%
Hidden Units h				
2	0.652	0.204	29.7%	4.7%
4	**0.612**	0.171	**30.5%**	**0.5%**
6	0.673	**0.148**	13.8%	3.1%
8	0.757	0.157	7.6%	5.7%
10	0.866	0.156	2.9%	22.9%
12	0.881	0.162	1.8%	29.9%
14	0.774	0.180	8.1%	16.7%
16	0.809	0.161	5.7%	16.4%

## Data Availability

Data are contained within the article.
